# PD-L1 Protein Expression Is Associated With Good Clinical Outcomes and Nomogram for Prediction of Disease Free Survival and Overall Survival in Breast Cancer Patients Received Neoadjuvant Chemotherapy

**DOI:** 10.3389/fimmu.2022.849468

**Published:** 2022-05-20

**Authors:** Li Chen, Shaolong Huang, Qiang Liu, Xiangyi Kong, Zhaohui Su, Mengliu Zhu, Yi Fang, Lin Zhang, Xingrui Li, Jing Wang

**Affiliations:** ^1^Department of Thyroid and Breast Surgery, Tongji Hospital, Tongji Medical College, Huazhong University of Science and Technology, Wuhan, China; ^2^Department of Breast Surgical Oncology, National Cancer Center/National Clinical Research Center for Cancer/Cancer Hospital, Chinese Academy of Medical Sciences and Peking Union Medical College, Beijing, China; ^3^Department of Thyroid and Breast, Burn and Plastic Surgery, Tongren City People’s Hospital, Tongren, China; ^4^School of Public Health, Southeast University, Nanjing, China; ^5^Melbourne School of Population and Global Health, The University of Melbourne, VIC, Australia; ^6^Centre of Cancer Research, Victorian Comprehensive Cancer Centre, Melbourne, VIC, Australia; ^7^School of Population Medicine and Public Health, Chinese Academy of Medical Sciences and Peking Union Medical College, Beijing, China

**Keywords:** breast cancer, immune checkpoint inhibitors, programmed cell death 1 (PD-1), programmed cell death ligand-1 (PD-L1), neoadjuvant chemotherapy

## Abstract

**Objective:**

This study aims to investigate the potential prognostic significance of programmed death ligand-1 (PD-L1) protein expression in tumor cells of breast cancer patients received neoadjuvant chemotherapy (NACT).

**Methods:**

Using semiquantitative immunohistochemistry, the PD-L1 protein expression in breast cancer tissues was analyzed. The correlations between PD-L1 protein expression and clinicopathologic characteristics were analyzed using Chi-square test or Fisher’s exact test. The survival curve was stemmed from Kaplan-Meier assay, and the log-rank test was used to compare survival distributions against individual index levels. Univariate and multivariate Cox proportional hazards regression models were accessed to analyze the associations between PD-L1 protein expression and survival outcomes. A predictive nomogram model was constructed in accordance with the results of multivariate Cox model. Calibration analyses and decision curve analyses (DCA) were performed for the calibration of the nomogram model, and subsequently adopted to assess the accuracy and benefits of the nomogram model.

**Results:**

A total of 104 breast cancer patients received NACT were enrolled into this study. According to semiquantitative scoring for IHC, patients were divided into: low PD-L1 group (61 cases) and high PD-L1 group (43 cases). Patients with high PD-L1 protein expression were associated with longer disease free survival (DFS) (mean: 48.21 months vs. 31.16 months; P=0.011) and overall survival (OS) (mean: 83.18 months vs. 63.31 months; P=0.019) than those with low PD-L1 protein expression. Univariate and multivariate analyses indicated that PD-L1, duration of neoadjuvant therapy, E-Cadherin, targeted therapy were the independent prognostic factors for patients’ DFS and OS. Nomogram based on these independent prognostic factors was used to evaluate the DFS and OS time. The calibration plots shown PD-L1 based nomogram predictions were basically consistent with actual observations for assessments of 1-, 3-, and 5-year DFS and OS time. The DCA curves indicated the PD-L1 based nomogram had better predictive clinical applications regarding prognostic assessments of 3- and 5-year DFS and OS, respectively.

**Conclusion:**

High PD-L1 protein expression was associated with significantly better prognoses and longer DFS and OS in breast cancer patients. Furthermore, PD-L1 protein expression was found to be a significant prognostic factor for patients who received NACT.

## Introduction

Breast cancer (BC) is one of the most common aggressive human cancers in the clinical setting, and is the primary cause of morbidity and mortality in women across the world (1). Due to a dearth of research, previously, breast cancer has been wrongly categorized as a non-immunogenic cancer (2). However, accumulating evidence continues to indicate otherwise, ranging from the presence of adaptive immune response that regulates breast cancer growth to the observance of a large number of heavy tumors infiltrated immune cells (3). Currently, treatment strategies for breast cancer, ranging from operation, chemotherapy, radiotherapy, endocrine therapy to targeted therapy, are commonly used in clinic and have definite curative effect (4, 5). Nowadays, cancer immunotherapy has become the new pillar of breast cancer treatment, and its use is approved for integrating with chemotherapy for first-line therapy (6, 7).

Immune checkpoint inhibitors (ICI), for instance, programmed cell death 1 (PD-1), programmed cell death ligand-1 (PD-L1), cytotoxic T lymphocyte antigen 4 (CTLA-4) have shown notably promise for the treatment of various cancers (8). And the crucial changes in these immune cells in cancerous tumors may contribute to the forecasting of the prognosis of cancer patients. As is known to all, PD-1 and PD-L1 are momentous immune checkpoint components that essentially regulate the function of tumor-infiltrating lymphocytes (TILs) and tumor cells (9). PD-1 is a type I transmembrane glycoprotein belonging to the immunoglobulin CD28 superfamily (10). As a cell surface receptor, PD-1 is predominantly expressed on the cell surface of T cells, B cells, Natural killer (NK) cells, monocytes, dendritic cells (DCs), tumor cells (11). Furthermore, PD-1 can negatively regulate the activity of T cells *via* interacting with its ligands PD-L1 (B7-H1) and PD-L2 (B7-DC) expressing on immune cells and tumor cells at some steps of the immune response (12). PD-L1 is a type I transmembrane glycoprotein, and is mainly expressed on the cell surface of T cells, B cells, DCs, macrophages, and tumor cells (13). Through binding PD-1, PD-L1 induces activated anti-tumor T cells, and plays a major role in the inhibition of T cell-mediated immune response (14). PD-1 and PD-L1 interact with each other attenuating local immune responses and shielding tumor cells from T cell-mediated killing (15).

Recently, the PD-1/PD-L1 pathway regulates tumor microenvironment through the induction and maintenance of immune tolerance, and PD-1 and PD-L1 have been proved to be promising targets for the treatment of a large number of tumor types, such as hepatocellular carcinoma (HCC), pancreatic cancer, gastric neuroendocrine carcinomas, thymic carcinoma, non-small-cell lung cancer (NSCLC) (16–20). One study from Europe found that PD-1 positive immune cells correlated with longer disease free survival time in triple negative breast cancer (TNBC), and the density of TILs was notably associated with PD-1 and PD-L1 expression in immune cells (21). Research also indicated that PD-L1 was markedly enriched in basal-like breast cancer and was correlated with infiltrating lymphocytes, and improved disease-specific survival time in ER-negative disease (22). Moreover, another study performed that a positive correlation of CD8-positive T cells and PD-L1 expression in HER2-positive breast carcinoma patients, and might predict a favorable survival outcome (23).

However, the role of PD-L1 in breast cancer oncogenesis and treatment is still quite obscure currently. Against this backdrop, the potential clinical significance of PD-L1 protein expression in breast cancer patients who underwent neoadjuvant chemotherapy (NACT) have been very scarcely studied. Here, we aimed to identify the potential predictive and prognostic value of PD-L1 protein expression in breast cancer received NACT, and evaluate this would be useful as a predictor for estimating treatment response.

## Materials and Methods

### Patients and Samples

In the current study, we retrospectively enrolled 104 patients with breast cancer for whom formalin fixed paraffin embedded (FFPE) tissue specimens and 65 FFPE matching non-neoplastic background tissues had been handed in for safekeeping in Cancer Hospital Chinese Academy of Medical Sciences in China. All enrolled patients had complete clinical and demographic data from medical records, and were confirmed by histopathology as breast carcinoma. After NACT treatment, all patients have already undergone relevant surgeries, operations such as mastectomy and breast-conserving procedures.

### Ethical Approval and Informed Consent

The study involving human participants was reviewed and approved by the ethics committee of Cancer Hospital Chinese Academy of Medical Sciences. All processes performed in studies involving human participants were consistent with the standards of the institutional research committee and with the declaration of 1964 Helsinki as well as its later amendments or comparable ethical standards. The patients provided their written informed consent to participate in this study.

### Immunohistochemistry (IHC)

All patients underwent curative operation involving a mastectomy or breast-conserving surgery with axillary lymph node dissection (ALND) after NACT. The breast cancer tissues and adjacent normal breast tissues were fixed in methanol, embedded in paraffin, sectioned, and performed immunohistochemical analyses. According to the instructions of the manufacturer, the immunohistochemical staining was performed using a two-step kit, details of which have been described in our previously study (24). The PD-L1 levels were detected using the primary monoclonal antibody directed against PD-L1 (GB14132, dilution 1:500, Servicebio, China).

### Assessment of PD-L1 Protein Expression

Patients’ PD-L1 protein expressions were measured by a semiquantitative scoring method. The arrays were scanned by the Aperio Image Scope system (Leica Biosystems, United States). Histological analyses were performed by combining the density and intensity of positive staining cells. The density classification of positive cells was itemized here below: 1) 0, the number of positive cells < 5%; 2) 1, the number of positive cells 5-25%; 3) 2, the number of positive cells 26-50%; 4) 3, the number of positive cells 51-75%; 5) 4, the number of positive cells > 75%. The intensity of positive staining cells was itemized here below: 1) 0, absence of staining; 2) 1, light yellow staining; 3) 2, brownish yellow staining; 4) 3, brownish brown staining. The immunoreactivity of the PD-L1 proteins were scored on the basis of the intensity and density of positive stained cells, and the scores were calculated and assessed by two independent investigators. All specimens were examined and evaluated by two investigators blinded to the clinical information of the patients.

### Follow-Up

All enrolled patients had routine follow-ups (e.g., outpatient, inpatient, or telephone consultations). Follow-up evaluations were performed every 3 months for the first to the second year after receiving the operation, every 6 months for the third to the fifth year after the operation, then yearly thereafter. disease free survival (DFS) was defined as the time from surgery to progression with regard to the distant disease metastasis, death from any cause or last follow-up. Overall survival (OS) was defined as the time from surgery to the date of death from any cause or last follow-up.

### Statistical Analysis

All statistical analyses were performed using the SPSS software (version 17.0; SPSS Inc., Chicago, IL, USA), GraphPad Prism software (version 8.0; GraphPad Inc., La Jolla, CA, USA), and R (version 3.6.0; Vienna, Austria. URL: http://www.R-project.org/). The correlations between PD-L1 protein expression and clinicopathologic characteristics were presented as absolute values and percentages (%), and tested using the Chi-square test or Fisher’s exact test. The survival curve was developed utilizing the Kaplan-Meier method, the log-rank test was used to compare survival distributions of individual index levels. Univariate and multivariate Cox proportional hazards regression models were accessed to analyze the associations between PD-L1 protein expression and survival outcomes. A predictive nomogram model was constructed in accordance with the results of the multivariate Cox model. Calibration analyses and decision curve analyses (DCA) were performed for the calibration of the nomogram model, and subsequently adopted to assess the accuracy and benefits of the nomogram model. Two-sided P values less than 0.05 were considered statistically significant.

## Results

### PD-L1 Protein Expression in Breast Cancer Tissues and Non-Neoplastic Background Tissues Breast Tissues

PD-L1 was mainly expressed on the cytoplasm or membranous of tumor cells. According to the semiquantitative scoring method for IHC scores, we chose the median value of the IHC scores as the cutoff value. And these patients were subsequently divided into two groups, i.e., low PD-L1 group (61 cases) vs high PD-L1 group (43 cases). The **Figure S1** shown the different expression status of PD-L1 by IHC.

### Association Between PD-L1 Protein Expression With the Patients’ Characteristics in the Study

All cases were female, and the median age was 46 years ranging 27 to 73 years old. In terms of diagnosis, prior to NACT, 3 patients (2.9%) were diagnosed with stage I breast cancer, 39 patients (37.5%) were stage II, and 62 patients (59.6%) were stage III. Post operation, 2 patients (3.8%) were diagnosed with stage Tis/T0, 16 patients (15.4%) were stage I, 38 patients (36.5%) were stage II, and 48 patients (46.2%) were stage III. After surgery, 79 (76.0%) cases received radiotherapy, 60 (57.7%) cases had undergone endocrine therapy, 32 (30.8%) cases received targeted therapy, and 74 (71.2%) cases were undergoing chemotherapy. The basic clinicopathological features of the patients could be found in **Table 1**. PD-L1 was related to ABO blood type (P=0.019).

**Table 1:** Patients’ characteristics for all patients according to programmed cell death ligand-1 (PD-L1).

n	level	Low PD-L1	High PD-L1	P
		61	43	
Age (%) (years)	<46	31 (50.8)	17 (39.5)	0.349
	≥46	30 (49.2)	26 (60.5)	
Family history (%)	No	49 (80.3)	31 (72.1)	0.456
	Yes	12 (19.7)	12 (27.9)	
Menarche age (%) (year)	<14	25 (41.0)	14 (32.6)	0.504
	≥14	36 (59.0)	29 (67.4)	
Menopause (%)	No	40 (65.6)	24 (55.8)	0.422
	Yes	21 (34.4)	19 (44.2)	
ABO blood type (%)	A	16 (26.2)	12 (27.9)	0.019
	B	26 (42.6)	8 (18.6)	
	O	15 (24.6)	13 (30.2)	
	AB	4 (6.6)	10 (23.3)	
Tumor site (%)	Right	27 (44.3)	18 (41.9)	0.966
	Left	34 (55.7)	25 (58.1)	
Clinical T stage (%)	T1	7 (11.5)	8 (18.6)	0.483
	T2	32 (52.5)	25 (58.1)	
	T3	10 (16.4)	4 (9.3)	
	T4	12 (19.7)	6 (14.0)	
Clinical N stage (%)	N0	10 (16.4)	6 (14.0)	0.105
	N1	16 (26.2)	19 (44.2)	
	N2	27 (44.3)	10 (23.3)	
	N3	8 (13.1)	8 (18.6)	
Clinical TNM stage (%)	I	2 (3.3)	1 (2.3)	0.493
	II	20 (32.8)	19 (44.2)	
	III	39 (63.9)	23 (53.5)	
Type of surgery (%)	Mastectomy	54 (88.5)	34 (79.1)	0.298
	Breast-conserving surgery	7 (11.5)	9 (20.9)	
Pathological Tumor size (%)	≤2cm	26 (42.6)	19 (44.2)	0.316
	>2 and <5cm	29 (47.5)	23 (53.5)	
	≥5cm	6 (9.8)	1 (2.3)	
Histologic type (%)	Noninvasive carcinoma	4 (6.6)	0 (0.0)	0.232
	Invasive nonspecific carcinoma	57 (93.4)	43 (100.0)	
Histologic grade (%)	I	6 (9.8)	0 (0.0)	0.059
	II	34 (55.7)	31 (72.1)	
	III	21 (34.4)	12 (27.9)	
Pathological T stage (%)	Tis/T0	3 (4.9)	1 (2.3)	0.423
	T1	24 (39.3)	17 (39.5)	
	T2	26 (42.6)	23 (53.5)	
	T3	1 (1.6)	1 (2.3)	
	T4	7 (11.5)	1 (2.3)	
Pathological N stage (%)	N0	24 (39.3)	7 (16.3)	0.064
	N1	14 (23.0)	13 (30.2)	
	N2	8 (13.1)	11 (25.6)	
	N3	15 (24.6)	12 (27.9)	
Pathological TNM stage (%)	Tis/T0	2 (3.3)	0 (0.0)	0.121
	I	13 (21.3)	3 (7.0)	
	II	21 (34.4)	17 (39.5)	
	III	25 (41.0)	23 (53.5)	
Postoperative radiotherapy (%)	No	18 (29.5)	7 (16.3)	0.186
	Yes	43 (70.5)	36 (83.7)	
Postoperative endocrine therapy (%)	No	28 (45.9)	16 (37.2)	0.495
	Yes	33 (54.1)	27 (62.8)	
Targeted therapy (%)	No	42 (68.9)	30 (69.8)	1.000
	Yes	19 (31.1)	13 (30.2)	
Postoperative chemotherapy (%)	No	22 (36.1)	8 (18.6)	0.086
	Yes	39 (63.9)	35 (81.4)	

### Association Between PD-L1 Protein Expression With the Patients’ Pathology Parameters in the Study

Before NACT, patients with Luminal A were 8 cases (7.7%), Luminal B HER2 (+) were 14 cases (13.5%), Luminal B HER2 (-) were 35 cases (33.7%), HER2-enriched were 15 cases (14.4%), triple-negative were 32 cases (30.8%). After operation, patients with Luminal A were 17 cases (16.3%), Luminal B HER2 (+) were 9 cases (8.7%), Luminal B HER2 (-) were 23 cases (22.1%), HER2-enriched were 18 cases (17.3%), triple-negative were 37 cases (35.6%). However, no significant correlations between PD-L1 protein expression and pathology parameters were found (P>0.05). The detail information was shown in **Table 2**.

**Table 2:** Patients’ pathology parameters for all patients according to programmed cell death ligand-1 (PD-L1).

n	level	Low PD-L1	High PD-L1	P
		61	43	
**Core needle biopsy**				
Molecular subtype (%)	Luminal A	3 (4.9)	5 (11.6)	0.561
	Luminal B HER2+	7 (11.5)	7 (16.3)	
	Luminal B HER2-	22 (36.1)	13 (30.2)	
	HER2 enriched	8 (13.1)	7 (16.3)	
	Triple negative	21 (34.4)	11 (25.6)	
ER (%)	0-25%	29 (47.5)	19 (44.2)	0.617
	26-50%	4 (6.6)	5 (11.6)	
	51-75%	4 (6.6)	1 (2.3)	
	76-100%	24 (39.3)	18 (41.9)	
PR (%)	0-25%	39 (63.9)	26 (60.5)	0.701
	26-50%	4 (6.6)	4 (9.3)	
	51-75%	5 (8.2)	6 (14.0)	
	76-100%	13 (21.3)	7 (16.3)	
HER2 (%)	Negative	47 (77.0)	29 (67.4)	0.388
	Positive	14 (23.0)	14 (32.6)	
Ki-67 (%)	0-25%	22 (36.1)	17 (39.5)	0.786
	26-50%	23 (37.7)	16 (37.2)	
	51-75%	11 (18.0)	5 (11.6)	
	76-100%	5 (8.2)	5 (11.6)	
**Postoperative pathology**				
Molecular subtype (%)	Luminal A	11 (18.0)	6 (14.0)	0.535
	Luminal B HER2+	4 (6.6)	5 (11.6)	
	Luminal B HER2-	14 (23.0)	9 (20.9)	
	HER2 enriched	8 (13.1)	10 (23.3)	
	Triple negative	24 (39.3)	13 (30.2)	
ER (%)	0-25%	32 (52.5)	22 (51.2)	0.969
	26-50%	4 (6.6)	2 (4.7)	
	51-75%	3 (4.9)	2 (4.7)	
	76-100%	22 (36.1)	17 (39.5)	
PR (%)	0-25%	48 (78.7)	31 (72.1)	0.748
	26-50%	5 (8.2)	5 (11.6)	
	51-75%	3 (4.9)	4 (9.3)	
	76-100%	5 (8.2)	3 (7.0)	
HER2 (%)	Negative	49 (80.3)	31 (72.1)	0.456
	Positive	12 (19.7)	12 (27.9)	
Ki-67 (%)	0-25%	29 (47.5)	21 (48.8)	0.952
	26-50%	15 (24.6)	12 (27.9)	
	51-75%	9 (14.8)	5 (11.6)	
	76-100%	8 (13.1)	5 (11.6)	
AR (%)	0-25%	54 (88.5)	41 (95.3)	0.604
	26-50%	1 (1.6)	0 (0.0)	
	51-75%	2 (3.3)	1 (2.3)	
	76-100%	4 (6.6)	1 (2.3)	
CK5/6 (%)	Negative	44 (72.1)	31 (72.1)	1.000
	Positive	17 (27.9)	12 (27.9)	
E-cad (%)	Negative	12 (19.7)	12 (27.9)	0.456
	Positive	49 (80.3)	31 (72.1)	
EGFR (%)	Negative	35 (57.4)	22 (51.2)	0.669
	Positive	26 (42.6)	21 (48.8)	
P53 (%)	0-25%	42 (68.9)	27 (62.8)	0.530
	26-50%	10 (16.4)	6 (14.0)	
	51-75%	9 (14.8)	9 (20.9)	
	76-100%	0 (0.0)	1 (2.3)	
TOP2A (%)	0-25%	38 (62.3)	29 (67.4)	0.538
	26-50%	14 (23.0)	9 (20.9)	
	51-75%	9 (14.8)	4 (9.3)	
	76-100%	0 (0.0)	1 (2.3)	
Lymph vessel invasion (%)	Negative	38 (62.3)	24 (55.8)	0.645
	Positive	23 (37.7)	19 (44.2)	
Neural invasion (%)	Negative	47 (77.0)	34 (79.1)	0.996
	Positive	14 (23.0)	9 (20.9)	

ER, estrogen receptor; PR, progesterone receptor; HER2, Human Epidermal Growth Factor Receptor 2; AR, androgen receptor; E-cad, E-Cadherin; EGFR, epidermal growth factor receptor; TOP2A, Topoisomerase II-α.

### Association Between PD-L1 Protein Expression With the Patients’ Chemotherapy in the Study

All patients were received NACT, and the effect of chemotherapy was determined after two cycles. The clinical response was assessed according to the Response Evaluation Criteria in Solid Tumors (RECIST) guidelines (25). 104 patients responded to NACT, including 60 cases (57.7%) partial responses (PRs), 43 cases achieved stable disease (SD) and one case had progressive disease (PD). The pathological response to chemotherapy was assessed by Miller-Payne grade (MPG) (a five-point histological grading system) (26). Nine patients (8.7%) were Grade 1 response, 42 patients (40.4%) were Grade 2 response, 48 patients (46.2%) were Grade 3 response, one patient (1.0%) was Grade 4 response, 4 patients (3.8%) were Grade 5 response. However, no significant correlations between PD-L1 protein expression and clinical response and pathological response were found (P>0.05). A summary of the patients’ chemotherapy results could be found in **Table 3**.

**Table 3:** Patients’ chemotherapy for all patients according to programmed cell death ligand-1 (PD-L1).

n	level	Low PDL-1	High PD-L1	P
		61	43	
Neoadjuvant chemotherapy regimen (%)	AC/ACF	3 (4.9)	1 (2.3)	0.754
	CT/ACT	7 (11.5)	3 (7.0)	
	AT	28 (45.9)	25 (58.1)	
	TP	13 (21.3)	8 (18.6)	
	Others	10 (16.4)	6 (14.0)	
Duration of neoadjuvant therapy (%)	<6	16 (26.2)	18 (41.9)	0.144
	≥6	45 (73.8)	25 (58.1)	
Response (%)	PR	38 (62.3)	22 (51.2)	0.326
	SD	22 (36.1)	21 (48.8)	
	PD	1 (1.6)	0 (0.0)	
MPG (%)	1	7 (11.5)	2 (4.7)	0.568
	2	24 (39.3)	18 (41.9)	
	3	26 (42.6)	22 (51.2)	
	4	1 (1.6)	0 (0.0)	
	5	3 (4.9)	1 (2.3)	
Postoperative chemotherapy (%)	No	22 (36.1)	8 (18.6)	0.086
	Yes	39 (63.9)	35 (81.4)	
Postoperative chemotherapy regimen (%)	AC/ACF	7 (11.5)	2 (4.7)	0.068
	CT/ACT	1 (1.6)	5 (11.6)	
	AT	6 (9.8)	3 (7.0)	
	TP	9 (14.8)	8 (18.6)	
	Others	16 (26.2)	17 (39.5)	
	NO	22 (36.1)	8 (18.6)	
Postoperative chemotherapy times (%)	<4	36 (59.0)	12 (27.9)	0.003
	≥4	25 (41.0)	31 (72.1)	

A, Anthracyclines; C, Cyclophosphamide; F, 5-Fluorouracil; T, Taxol; P, Platinum compounds.

### Association Between PD-L1 Protein Expression With the Patients’ Side Effects of Chemotherapy in the Study

The common adverse events (AEs) (any-grade) during the NACT period were gastrointestinal reactions (included decreased appetite, nausea, vomiting, diarrhea, mouth ulcers, alopecia, peripheral neurotoxicity), hematologic reactions (anemia, leukopenia, neutropenia, thrombocytopenia), myelosuppression, and hepatic dysfunction. However, no significant correlations between PD-L1 and side effects of chemotherapy were found (P>0.05). The side effects of chemotherapy experienced by the patients could be found in **Table 4**.

**Table 4:** Patients’ side effects of chemotherapy for all patients according to programmed cell death ligand-1 (PD-L1).

n	level	Low PD-L1	High PD-L1	P
		61	43	
Decreased appetite (%)	No	10 (16.4)	7 (16.3)	1.000
	Yes	51 (83.6)	36 (83.7)	
Nausea (%)	No	7 (11.5)	4 (9.3)	0.975
	Yes	54 (88.5)	39 (90.7)	
Vomiting (%)	No	32 (52.5)	18 (41.9)	0.386
	Yes	29 (47.5)	25 (58.1)	
Diarrhea (%)	No	57 (93.4)	40 (93.0)	1.000
	Yes	4 (6.6)	3 (7.0)	
Mouth ulcers (%)	No	60 (98.4)	42 (97.7)	1.000
	Yes	1 (1.6)	1 (2.3)	
Alopecia (%)	No	29 (47.5)	19 (44.2)	0.890
	Yes	32 (52.5)	24 (55.8)	
Peripheral neurotoxicity (%)	No	48 (78.7)	39 (90.7)	0.173
	Yes	13 (21.3)	4 (9.3)	
Anemia (%)	Grade 0	34 (55.7)	21 (48.8)	0.621
	Grade 1-2	27 (44.3)	22 (51.2)	
Leukopenia (%)	Grade 0	12 (19.7)	12 (27.9)	0.502
	Grade 1-2	35 (57.4)	20 (46.5)	
	Grade 3-4	14 (23.0)	11 (25.6)	
Neutropenia (%)	Grade 0	8 (13.1)	12 (27.9)	0.157
	Grade 1-2	25 (41.0)	16 (37.2)	
	Grade 3-4	28 (45.9)	15 (34.9)	
Thrombocytopenia (%)	Grade 0	49 (80.3)	30 (69.8)	0.313
	Grade 1-2	12 (19.7)	13 (30.2)	
Gastrointestinal reaction (%)	Grade 0	7 (11.5)	5 (11.6)	0.701
	Grade 1-2	53 (86.9)	38 (88.4)	
	Grade 3-4	1 (1.6)	0 (0.0)	
Myelosuppression (%)	Grade 0	5 (8.2)	10 (23.3)	0.096
	Grade 1-2	20 (32.8)	11 (25.6)	
	Grade 3-4	36 (59.0)	22 (51.2)	
Hepatic dysfunction (%)	Grade 0	42 (68.9)	24 (55.8)	0.249
	Grade 1-2	19 (31.1)	19 (44.2)	

### Survival Analysis for PD-L1 Protein Expression

**Figure 1:**
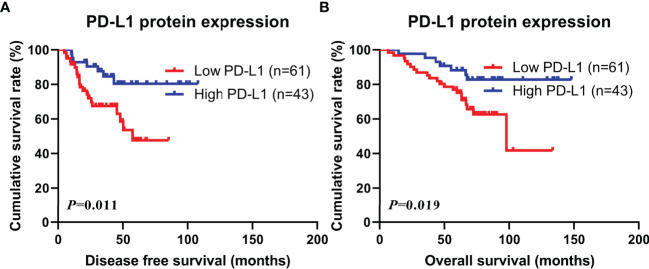
Kaplan-Meier curves for disease free survival (DFS) and overall survival (OS). **(A)** Kaplan-Meier curves for DFS for PD-L1 protein expression in tumor cells; **(B)** Kaplan-Meier curves for OS for PD-L1 protein expression in tumor cells.

Through PD-L1 protein expression on tumor cells, the DFS and OS were compared separately. The mean DFS and OS levels for patients in the low PD-L1 group were 31.16 months (range from 4.67 to 85.07 months) and 63.31 months (range from 6.43 to 133.60 months), while the mean DFS and OS values for patients in the high PD-L1 group were 48.21 months (range from 10.17 to 107.80 months) and 83.18 months (range from 14.47 to 148.00 months), respectively. Patients with high PD-L1 protein expression revealed significantly better DFS and OS than those with low PD-L1 protein expression (χ^2 =^ 6.440, P=0.011; χ^2 =^ 5.483, P=0.019; see in **Figures 1A, B**).

### Univariate and Multivariate Analyses for Disease Free Survival (DFS) and Overall Survival (OS)

According to the Cox proportional-hazards models for DFS, the univariate analysis suggested that PD-L1, tumor site, neoadjuvant chemotherapy regimen, duration of neoadjuvant therapy, pathology (ER, Ki-67, E-cad), postoperative endocrine therapy, and targeted therapy were related to the prognosis of breast cancer patients, however, the multivariate analysis found that only PD-L1, duration of neoadjuvant therapy, pathology E-cad, targeted therapy were the independent prognostic factors (**Table 5**).

**Table 5:** Univariate and multivariate Cox proportional hazards regression model for disease free survival (DFS) and overall survival (OS).

Parameters	Level	DFS				OS				
		Univariate analysis		Multivariate analysis		Univariate analysis		Multivariate analysis	
		Hazard ratio (95%CI)	P	Hazard ratio (95%CI)	P	Hazard ratio (95%CI)	P	Hazard ratio (95%CI)	P
PD-L1	Low expression	1 (Reference)	0.027	1 (Reference)	0.011	1 (Reference)	0.042	1 (Reference)	0.001
	High expression	0.648(0.442-0.952)		0.605(0.411-0.891)		0.573(0.335-0.979)		0.528(0.359-0.777)	
Age	<46	1 (Reference)	0.064			1 (Reference)	0.628		
	≥46	1.966(0.961-4.019)				1.194(0.582-2.450)			
BMI	<23.63	1 (Reference)	0.130			1 (Reference)	0.023		
	≥23.63	1.595(0.871-2.918)				2.107(1.106-4.015)			
Family history	No	1 (Reference)	0.387			1 (Reference)	0.023		
	Yes	1.401(0.653-3.008)				2.445(1.133-5.279)			
Menopause	No	1 (Reference)	0.082			1 (Reference)	0.919		
	Yes	0.463(0.194-1.103)				0.958(0.420-2.183)			
Tumor site	Right	1 (Reference)	0.007			1 (Reference)	0.008		
	Left	2.065(1.219-3.497)				2.175(1.225-3.862)			
Clinical T stage	T1	1 (Reference)	0.231			1 (Reference)	0.132		
	T2+T3+T4	1.662(0.724-3.814)				1.990(0.813-4.870)			
Clinical N stage	N0	1 (Reference)	0.346			1 (Reference)	0.661		
	N1+N2+N3	0.508(0.124-2.076)				0.748(0.204-2.739)			
Clinical TNM stage	I	1 (Reference)	0.825			1 (Reference)	0.997		
	II+III	1.354(0.092-19.834)				1.005(0.064-15.808)			
Neoadjuvant chemotherapy regimen	AC/ACF/CT/ACT/AT	1 (Reference)	0.008			1 (Reference)	0.018		
	TP/Others	0.408(0.209-0.795)				0.425(0.209-0.862)			
Duration of neoadjuvant therapy	<6	1 (Reference)	0.003	1 (Reference)	0.002	1 (Reference)	0.007	1 (Reference)	0.002
	≥6	2.904(1.436-5.873)		1.998(1.285-3.105)		2.699(1.312-5.550)		1.973(1.291-3.016)	
Response	PR	1 (Reference)	0.306			1 (Reference)	0.685		
	SD+PD	0.747(0.428-1.305)				0.898(0.533-1.512)			
Type of surgery	Mastectomy	1 (Reference)	0.320			1 (Reference)	0.177		
	Breast-conserving surgery	0.660(0.291-1.497)				0.596(0.281-1.263)			
Pathological Tumor size	≤2cm	1 (Reference)	0.542			1 (Reference)	0.807		
	>2cm	1.530(0.390-6.001)				0.826(0.178-3.842)			
MPG	1+2	1 (Reference)	0.558			1 (Reference)	0.005	1 (Reference)	0.010
	3+4+5	1.209(0.641-2.280)				2.431(1.311-4.510)		1.984(1.174-3.353)	
Histologic type	Noninvasive carcinoma	1 (Reference)	0.720			1 (Reference)	0.621		
	Invasive nonspecific carcinoma	1.490(0.168-13.226)				1.697(0.209-13.807)			
Histologic grade	I	1 (Reference)	0.492			1 (Reference)	0.251		
	II+III	1.829(0.327-10.234)				0.354(0.060-2.090)			
Pathological T stage	T1	1 (Reference)	0.723			1 (Reference)	0.461		
	T2+T3+T4	1.313(0.292-5.906)				1.843(0.363-9.347)			
Pathological N stage	N0	1 (Reference)	0.373			1 (Reference)	0.748		
	N1+N2+N3	2.076(0.417-10.336)				1.244(0.329-4.705)			
Pathological TNM stage	I	1 (Reference)	0.091			1 (Reference)	0.055		
	II+III	0.235(0.044-1.258)				0.207(0.041-1.034)			
TLN	<24	1 (Reference)	0.686			1 (Reference)	0.146		
	≥24	1.119(0.649-1.930)				0.647(0.360-1.164)			
PLN	<2	1 (Reference)	0.479			1 (Reference)	0.030	1 (Reference)	0.000
	≥2	1.415(0.541-3.700)				2.769(1.105-6.937)		2.156(1.402-3.317)	
**Postoperative pathology**									
Molecular subtype	Luminal A/B HER2+/B HER2-	1 (Reference)	0.543			1 (Reference)	0.304		
	HER2 enriched/Triple negative	1.539(0.384-6.172)				2.322(0.466-11.569)			
ER	0-25%	1 (Reference)	0.023			1 (Reference)	0.001		
	26-100%	6.765(1.296-35.322)				25.813(3.734-178.431)			
PR	0-25%	1 (Reference)	0.738			1 (Reference)	0.216		
	26-100%	0.889(0.447-1.770)				1.609(0.757-3.419)			
HER2	Negative	1 (Reference)	0.485			1 (Reference)	0.514		
	Positive	0.698(0.254-1.919)				0.718(0.265-1.945)			
Ki-67	0-25%	1 (Reference)	0.020			1 (Reference)	0.015		
	26-100%	2.862(1.184-6.921)				2.786(1.219-6.368)			
AR	0-25%	1 (Reference)	0.619			1 (Reference)	0.072		
	26-100%	1.261(0.505-3.150)				0.402(0.149-1.085)			
CK5/6	Negative	1 (Reference)	0.292			1 (Reference)	0.006		
	Positive	0.630(0.266-1.489)				0.300(0.128-0.704)			
E-cad	Negative	1 (Reference)	0.022	1 (Reference)	0.007	1 (Reference)	0.000	1 (Reference)	0.014
	Positive	2.415(1.133-5.146)		1.934(1.196-3.126)		5.356(2.250-12.749)		1.984(1.147-3.431)	
EGFR	Negative	1 (Reference)	0.303			1 (Reference)	0.009		
	Positive	1.595(0.656-3.877)				3.560(1.368-9.264)			
P53	0-25%	1 (Reference)	0.291			1 (Reference)	0.014		
	26-100%	1.443(0.730-2.852)				2.270(1.183-4.357)			
TOP2A	0-25%	1 (Reference)	0.381			1 (Reference)	0.720		
	26-100%	0.672(0.276-1.635)				0.870(0.405-1.867)			
Lymph vessel invasion	Negative	1 (Reference)	0.767			1 (Reference)	0.757		
	Positive	0.903(0.460-1.772)				1.119(0.549-2.284)			
Neural invasion	Negative	1 (Reference)	0.101			1 (Reference)	0.405		
	Positive	1.997(0.873-4.565)				1.392(0.640-3.029)			
Postoperative chemotherapy	Negative	1 (Reference)	0.291			1 (Reference)	0.004		
	Positive	1.511(0.703-3.247)				3.244(1.445-7.280)			
Postoperative radiotherapy	Negative	1 (Reference)	0.573			1 (Reference)	0.678		
	Positive	0.801(0.370-1.735)				1.178(0.543-2.555)			
Postoperative endocrine therapy	Negative	1 (Reference)	0.012			1 (Reference)	0.000		
	Positive	0.408(0.202-0.823)				0.192(0.095-0.387)			
Targeted therapy	Negative	1 (Reference)	0.003	1 (Reference)	0.000	1 (Reference)	0.000	1 (Reference)	0.000
	Positive	3.898(1.602-9.486)		3.037(1.976-4.670)		6.576(2.565-16.860)		3.322(2.192-5.036)	

MPG, Miller-Payne grade; TLN, total lymph node; PLN, positive lymph node; ER, estrogen receptor, PR, progesterone receptor; HER2, Human Epidermal Growth Factor Receptor 2; AR, androgen receptor; E-cad, E-Cadherin; EGFR, epidermal growth factor receptor; TOP2A, Topoisomerase II-α

Through the Cox proportional-hazards models for OS, the univariate analysis indicated that PD-L1, BMI, family history, tumor site, neoadjuvant chemotherapy regimen, duration of neoadjuvant therapy, MPG, positive lymph node (PLN), pathology (ER, Ki-67, CK5/6, E-cad, EGFR, P53), postoperative chemotherapy, postoperative endocrine therapy, targeted therapy were related to the prognosis of breast cancer patients, however, the multivariate analysis found that only PD-L1, duration of neoadjuvant therapy, MPG, PLN, pathology E-cad, targeted therapy were the independent prognostic factors (**Table 5**).

As shown in **Table 5** univariate and multivariate analyses, the PD-L1 protein expression in tumor cells was related to favorable DFS (HR=0.648, 95%CI: 0.442-0.952, P=0.027; HR=0.605, 95%CI: 0.411-0.891, P=0.011) and OS (HR=0.573, 95%CI: 0.335-0.979, P=0.042; HR=0.528, 95%CI: 0.359-0.777, P=0.001) survival of breast cancer patients.

### Screening for Independent Predictors and Developing the Nomogram

Nomograms were supposed to be an uncomplicated tool to provide personality risk assessment for each patient (27). We constructed an effective and novel nomogram for individualized assessment of DFS and OS after NACT and surgery. In accordance with the independent prognostic factors identified in the Cox proportional-hazards models, a nomogram was developed to predict the DFS probability of breast cancer at 1-, 3-, and 5- year after radical surgery, and OS probability of breast cancer at 1-year, 3-year, 5-year, and 10-year after radical surgery.

**Figure 2:**
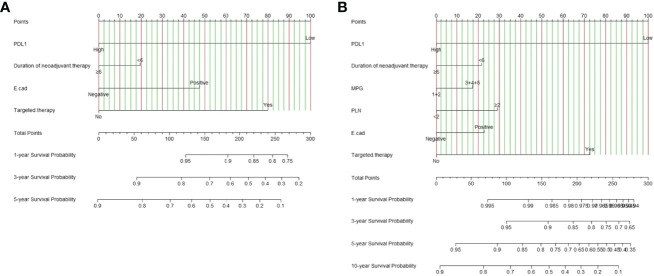
PD-L1-based nomogram for predicting disease free survival (DFS) and overall survival (OS). A straight upward line is drawn to determine the points for every predictor. The sum of these points is situated on the total points axis, and a straight downward line shows the 1-year, 3-year, 5-year DFS estimated rates and 1-year, 3-year, 5-year, 10-year OS estimated rates. **(A)** PD-L1 based nomogram for predicting disease free survival (DFS); **(B)** PD-L1 based nomogram for predicting and overall survival (OS). E-cad, E-Cadherin; PLN, positive axillary lymph node.

The nomogram for DFS had unique features, and integrated PD-L1, duration of neoadjuvant therapy, pathology E-cad, targeted therapy (**Figure 2A**). The nomogram for OS had distinguishing characteristics, including PD-L1, duration of neoadjuvant therapy, MPG, PLN, pathology E-cad, targeted therapy was an independent prognostic factor (**Figure 2B**).

### Calibration and Validation of the Nomogram

**Figure 3:**
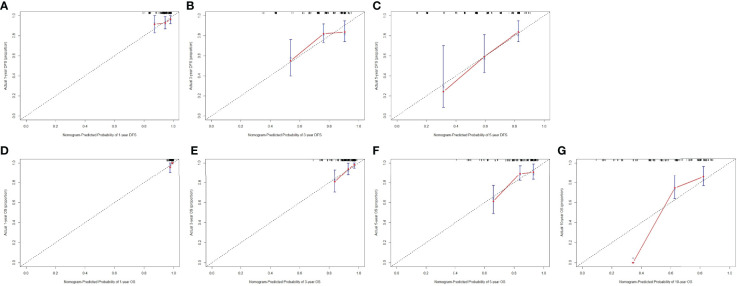
The calibration curves for predicting the 1-year, 3-year, 5-year DFS rate and 1-year, 3-year, 5-year, 10-year OS rates in patients with breast cancer who underwent NACT. The X-axis presents the nomogram-predicted probability of disease free survival (DFS) and overall survival (OS); the Y-axis shows the actual DFS and OS. **(A)** The calibration curves for predicting the 1-year DFS rate in patients with breast cancer; **(B)** The calibration curves for predicting the 3-year DFS rate in patients with breast cancer; **(C)** The calibration curves for predicting the 5-year DFS rate in patients with breast cancer; **(D)** The calibration curves for predicting the 1-year OS rate in patients with breast cancer; **(E)** The calibration curves for predicting the 3-year OS rate in patients with breast cancer; **(F)** The calibration curves for predicting the 5-year OS rate in patients with breast cancer; **(G)** The calibration curves for predicting the 10-year OS rate in patients with breast cancer.

Calibration curves (1000 bootstrap resamples) were established to check the concordance between the nomogram predicted and the actual probability of DFS and OS. The calibration plots for postoperative 1-year, 3-year, 5-year DFS survival indicated that PD-L1 based nomogram predictions were basically consistent with actual observations, especially in 5-year DFS survival (**Figures 3A–C**). The calibration plots for postoperative 1-year, 3-year, 5-year OS survival shown that PD-L1 based nomogram predictions were basically consistent with actual observations, especially in 3-year OS survival (**Figures 3D–F**). However, the calibration plots for postoperative 10-year OS survival revealed that PD-L1 based nomogram predictions were not well consistent with actual observations (**Figure 3G**).

### Clinical Use by Decision Curve Analysis (DCA)

**Figure 4:**
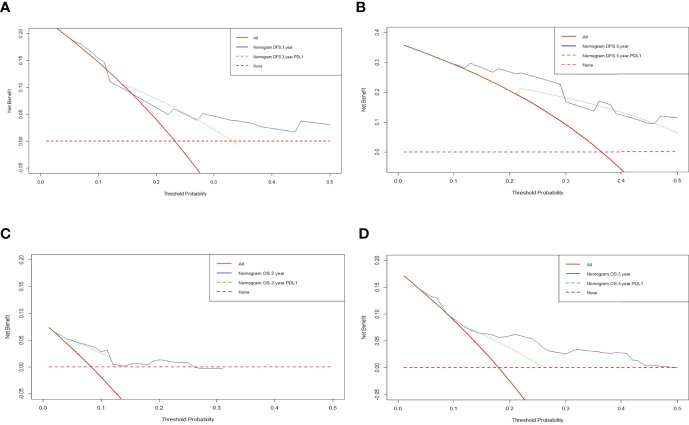
Decision curve analysis (DCA) of the nomograms and PD-L1 for predicting the disease free survival (DFS) and overall survival (OS). The X-axis represents threshold probability, and the Y-axis shows net benefit. The lines between the X-axis and the Y-axis display the benefit of different predictive variables. The red dotted line suggested that no patient has a poor prognosis, while the red line indicated that all patients have a poor prognosis. **(A)** DCA of the nomograms and PD-L1 for predicting the 3-year DFS; **(B)** DCA of the nomograms and PD-L1 for predicting the 5-year DFS; **(C)** DCA of the nomograms and PD-L1 for predicting the 3-year OS; **(D)** DCA of the nomograms and PD-L1 for predicting the 5-year OS.

The decision curve analysis (DCA) was conducted to evaluate the clinical usefulness of the DFS and OS nomogram by quantifying the net benefits at different threshold probabilities. DCA was performed to compare the clinical usability and benefits of the 3-year, 5-year DFS and OS nomogram with that of the PD-L1. The DCA curves indicated that the nomogram 3-year, 5-year DFS and OS had better predictive clinical application than PD-L1 (**Figure 4**).

## Discussion

There is growing evidence that the immune response status may be a critical determinant of influencing cancer progression and metastasis (28). It is universally acknowledged that anomalous immunosurveillance and immune escape of tumor cells play an important role in influencing antitumor immune response, aggressive growth and carcinogenesis of cancer cells (2, 29). Tumor tissue is not only composed of cancer cells, but also includes inflammatory cells, blood vessels, immune cells, fibrous tissue; and these components constitute a characteristic tumor immune microenvironment (TIME). Moreover, the TIME affects the prognosis and effectiveness of treatment, such as immunotherapy, and response to other treatments (30). That is to say, evaluating the TIME in each individual patient is helpful to predict the treatment response to different treatment patterns and anti-cancer drugs. In recent years, studies have been demonstrated that cancer immunotherapy is a major emerging treatment for breast cancer in clinical practice, following operation, chemotherapy, endocrine therapy, and radiotherapy (31).

The PD-1 and PD-L1 are two of the most critical biological inhibitors that allow cancer cells to escape host immunity. Blocking antibodies against PD-1 and PD-L1 can lead to local control and persistent response in patients with various tumors who are ineffective to standard treatment. The PD-1/PD-L1 pathway is also expected to effectively reverse the immunosuppression in tumors’ microenvironments (17). Thus, in light of the promising potential of the anti-PD-1 or anti-PD-L1 immunotherapy in cancer treatments (32), it is of vital importance to gain an in-depth and comprehensive understanding of the regulatory mechanisms of PD-1 and PD-L1 in carcinogenesis, progression, and metastasis. Previous studies have shown that PD-1 and PD-L1 protein expression were associated with the prognosis in different malignant tumors (33–35). However, due to significant limitations of these studies (e.g., Ahmed FS’s study (36), Wesolowski R’s study (37), and Loibl S’s study (38)), research into the potential significance of PD-1 and PD-L1 protein expression in breast cancer patients treated with NACT was often considered flawed or controversial. Therefore, in light of their research and practical significance, to bridge the research gap, the present study investigated the PD-L1 protein expression in post-NACT patients’ tumor tissues and examined the relationship between the PD-L1 protein expression and patients’ treatment efficacy.

The present study assessed how the protein expression of PD-L1 immune checkpoints affect the responses of breast cancer patients to NACT. By using IHC assay, we demonstrated that high PD-L1 protein expression at the protein level were related to better prognoses in breast cancer patients. Matikas A and associates found that PD-1 protein and gene expression seem to be promising prognostic factors in early breast cancer, and PD-L1 gene expression is a promising prognostic factor, especially in basal-like breast cancer (39, 40). Through the IHC score by semiquantitative scoring method, all patients were classified as two groups. High PD-L1 protein expression were associated with better prognosis and significantly longer DFS and OS, respectively. Patients with high PD-L1 protein expression had long-term DFS and OS survival, and PD-L1 protein expression was an independent prognostic factor. In Botti G’s study, PD-L1 protein expression was strongly correlated with better DFS, yet not associated with OS; and PD-L1 could be an important marker for prognostic stratification and planning immune checkpoint inhibitor treatment in TNBC patients (41). Other study shown that patients with PD-L1 positive expression in tumor cells had better good recurrence-free survival (RFS) and OS than those with PD-L1 negative expression in tumor cells; and PD-L1 expression was found to be an independent marker for favorable RFS and OS in TNBC patients (42). Another study on analyzing transcriptional data of breast cancer from TCGA, PD-1 and PD-L1 gene expression were associated with immune infiltration and immune checkpoints; and PD-1 expression was associated with favorable survival of breast cancer patients (43). These literatures were consistent with our study. However, in Asano Y’s study, high PD-1 and PD-L1 protein expression was associated with a poorer prognosis in breast cancer patient before undergoing NACT, and low PD-1 and PD-L1 protein expression in TNBC patients was be bound up with a higher pCR rate and significantly longer DFS (44). And Chen’s study indicated that high expression of PD-L1 had a bearing on worse survival in breast cancer patients after NACT, and was used as a prognostic marker in non-pCR patients (45). Thus, due to conflicting research findings in the literature, it is still unclear whether PD-L1 expression could accurately predict the prognosis of breast cancer. This might explain the results from our study: 1) the expression of PD-L1 might be related to TIL-mediated antitumor inflammatory response, and rather than tumor immune escape; 2) the expression of PD-L1 varied in different molecular types of breast cancer; 3) the expression of PD-L1 were at the protein level rather than at the mRNA level.

In this study, breast cancer patients were divided into low expression and high expression groups according to their IHC scores. We analyzed the relationship between PD-L1 with the patients’ characteristics, and the results revealed that PD-L1 was associated with ABO blood type. In Iwasaki K’s study, the PD-L1 expression was related to the ABO-I renal transplants when compared with those from ABO-identical/compatible transplants (46). Moreover, the results also indicated that no significant difference between PD-L1 and pathology parameters. Furthermore, our results also showed that there was no significant difference between PD-L1 and patients’ chemotherapy or side effects of chemotherapy.

The univariate and multivariate analyses revealed that PD-L1, duration of neoadjuvant therapy, pathology E-cad, targeted therapy were the independent prognostic factors for DFS; and PD-L1, duration of neoadjuvant therapy, MPG, PLN, pathology E-cad, targeted therapy were the independent prognostic factors for OS. Thus, we constructed a new nomogram based on the independent prognostic factors to evaluate the DFS and OS survival in breast cancer received NACT. And the calibration plots for postoperative 1-year, 3-year, 5-year DFS and OS survival shown that PD-L1 based nomogram predictions were basically consistent with actual observations, especially in 5-year DFS survival and 3-year OS survival. Moreover, the DCA curves indicated that the PD-L1 score-based nomogram offers prognostic assessment of 3-year, 5-year DFS and OS had better predictive clinical application after NACT, and might bring great benefits to clinical practice.

However, some limitations should be considered in our study. Firstly, this study is retrospective in nature, which means that it is vulnerable to potential selective bias. However, it is important to note that the retrospective research approach also ensures that the findings of our study are grounded in reality, as they capture and reflect the real-world experiences of actual breast cancer patients who underwent NACT in China. Secondly, it is possible that variations in the PD-L1 antibodies used, IHC scoring, and patient selection might have contributed to the high heterogeneity of the research findings. Thirdly, some potential important parameters related to clinical prognosis are not examined in the current study, and the constructed nomogram was developed based on limited independent factors. Moreover, the heterogeneity and molecular subtype of breast cancer also influence the PD-L1 expression. Finally, the nomogram was internally validated, and future studies focus on external validation using other populations.

## Conclusions

High PD-L1 protein expressions were associated with significantly better prognoses and longer DFS and OS in breast cancer patients. Furthermore, PD-L1 protein expression was found to be a significant prognostic factor for patients who received NACT. Our study also suggested that nomogram analyses could provide more accurate individualized predictions of DFS and OS survival in patients, and in turn, have the potential to assist clinicians to make more informed decisions in clinical practice.

## Data Availability Statement

The original contributions presented in the study are included in the article/**Supplementary Material**. Further inquiries can be directed to the corresponding authors.

## Ethics Statement

This study was approved by the ethics committee of Cancer Hospital Chinese Academy of Medical Sciences. The patients/participants provided their written informed consent to participate in this study.

## Author Contributions

Writing-original draft and writing-review and editing, LC, SH, and QL. Formal analysis, LC and XK. Data curation and investigation, LC and ZS. Methodology and supervision, YF and LZ. Resources, funding acquisition, and project administration, XL and JW. All authors contributed to the article and approved the submitted version.

## Funding

The work is partly supported by research grants from The National Nature Science Foundation of China (No. 81872160, No. 82072940, No. 82103047, No. 82102887 and No.81802676), Beijing Nature Science Foundation of China (No. 7191009, No. 7204293), and National Key R&D Program of China (No. 2018YFC1312100), the China National Key R&D (or Research and Development) Program (No. 2020AAA0105000 and 2020AAA0105004), the Special Research Fund for Central Universities, Peking Union Medical College (No. 3332019053), the Beijing Hope Run Special Fund of Cancer Foundation of China (No. LC2020L01, No. LC2019B03, No. LC2019L07), Wuhan Youth Cadre Project (2017zqnlxr01 and 2017zqnlxr02), Clinical Research Physician Program of Tongji Medical College, HUST (5001540018), the Golden Bridge Project Seed Fund of Beijing Association for Science and Technology (No. ZZ20004), the Chinese Young Breast Experts Research project (No. CYBER-2021-005), the 2021 Chaoyang District Social Development Science and Technology Plan Project (Medical and Health Field) (No. CYSF2115), the Beijing Xisike Clinical Oncology Research Foundation (No. Y-Young2021-0017), the XianSheng Clinical Research Special Fund of China International Medical Foundation (No. Z-2014-06-2103).

## Conflict of Interest

The authors declare that the research was conducted in the absence of any commercial or financial relationships that could be construed as a potential conflict of interest.

## Publisher’s Note

All claims expressed in this article are solely those of the authors and do not necessarily represent those of their affiliated organizations, or those of the publisher, the editors and the reviewers. Any product that may be evaluated in this article, or claim that may be made by its manufacturer, is not guaranteed or endorsed by the publisher.

## Supplementary Material

Supplementary Figure 1 Expression of PD-L1 in human breast cancer tissues.
