# Supplementary figures and images for: PD-L1 Protein Expression Is Associated With Good Clinical Outcomes and Nomogram for Prediction of Disease Free Survival and Overall Survival in Breast Cancer Patients Received Neoadjuvant Chemotherapy
Source: Front Immunol. 2022 May 20;13:849468. doi: 10.3389/fimmu.2022.849468 (PMC9163312; doi:10.3389/fimmu.2022.849468)

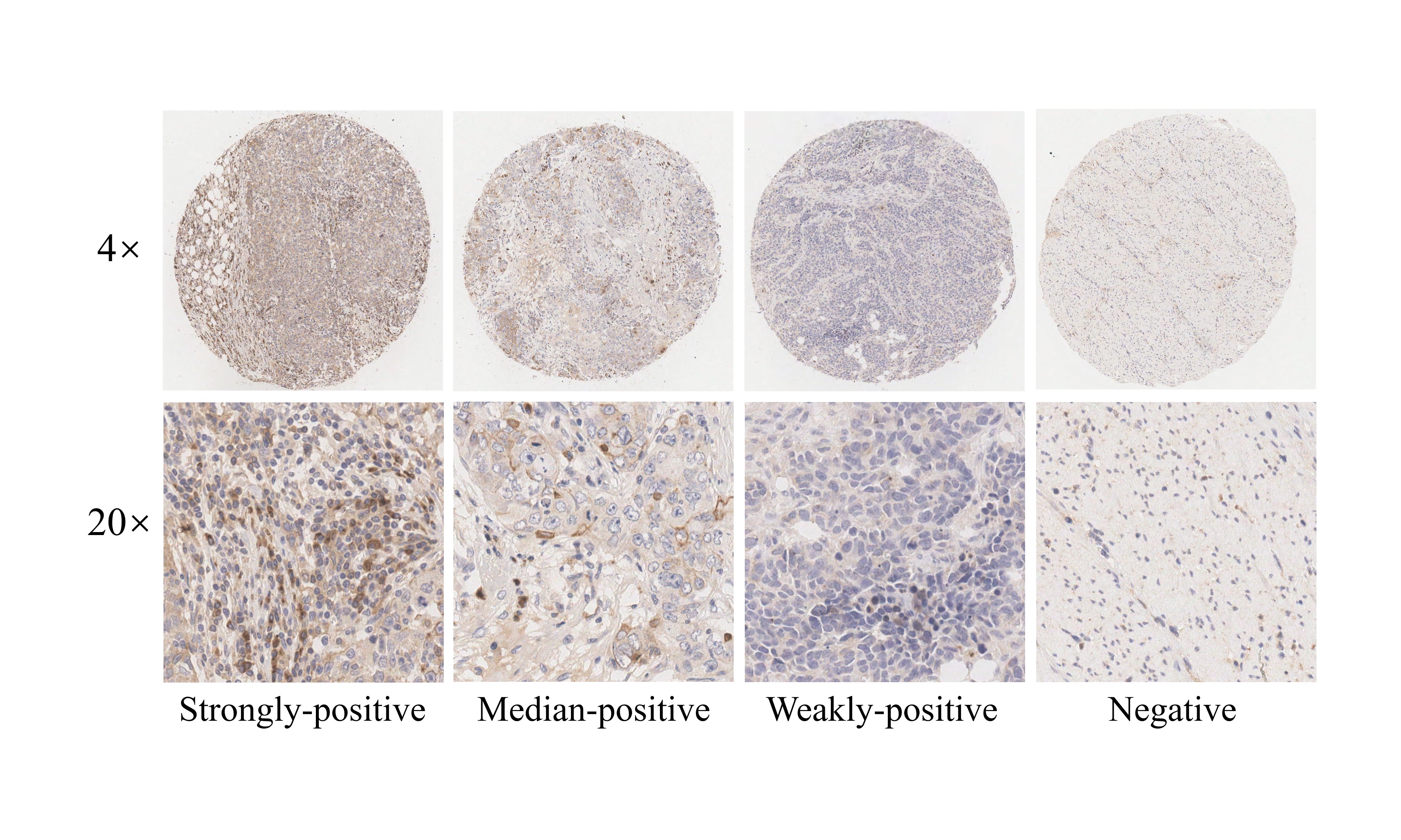

Supplement: Supplementary Figure 1 — Expression of PD-L1 in human breast cancer tissues. [file Image_1.jpeg]
